# Impact of microRNA-210 on wound healing among the patients with diabetic foot ulcer

**DOI:** 10.1371/journal.pone.0254921

**Published:** 2021-07-22

**Authors:** Sivakamasundari Pichu, Selvaraj Vimalraj, Vijay Viswanathan

**Affiliations:** 1 AU-KBC Research Center, Anna University MIT campus, Chromepet, Chennai, India; 2 Centre for Biotechnology, Anna University, Chennai, India; 3 Department of Pharmacology, Saveetha Dental College and Hospital, Saveetha Institute of Medical and Technical Sciences (SIMATS), Saveetha University, Chennai, Tamil Nadu, India; 4 Department of Genetics and Molecular Biology, Prof M. Viswanathan Diabetes Research Centre, MV Hospital for Diabetes, Royapuram, Chennai, India; Indiana University Purdue University at Indianapolis, UNITED STATES

## Abstract

**Aim:**

Diabetic foot ulcer (DFU) is a major concern in diabetes and its control requires in-depth molecular investigation. The present study aimed to screen the expression of microRNA-210 (miR-210) and its association in hypoxic pathway in DFU patients.

**Methods:**

The study consists of 3 groups of circulation samples (50 in each group of: healthy volunteers, T2DM and T2DM with DFU) and 2 groups of tissue samples (10 in each group of: control and T2DM with DFU). Expression of miR-210 and hypoxia inducible factor-1 alpha (HIF-1α), and its responsive genes such as VEGF, TNF-α, IL-6, BCl2, Bax and Caspase 3 were analyzed by RT-PCR, Western blot and ELISA analyses.

**Results:**

The HIF-1α expression decreased in DFU patients with increased miR-210 expression in both circulation and tissue biopsies. The circulatory IL-6 and inflammatory gene TNF-α expression was increased in DFU compared to healthy controls and T2DM subjects. Further, we found there was no alteration in the angiogenic marker, VEGF expression. In comparison, anti-apoptotic BCl2 was decreased and Bax and Caspase 3 was increased in DFU tissues relative to control.

**Conclusions:**

The study showed that there was an inverse relationship between miR-210 and HIF-1α expression in patients with DFU, indicating that miR-210 may regulate the expression of the hypoxic gene.

## 1. Introduction

Diabetes mellitus (DM) has been one of the most prevalent medical illnesses, presenting a huge global health threat. More than a third of diabetics experience significant complications. The basic causes for diabetic complications remain important to better understand and establish new and enhanced treatment approaches for these chronic conditions. Several pathogenic stimulants, including hyperlipidemia, hyperglycemia, advanced glycation end products, inflammatory cytokines/chemokines and growth factors have been linked to an increased risk of these problems in diabetics [1, 2]. The global health concern and recent increased research indicates that enhanced hypoxic conditions and cellular reaction are important factors in diabetic foot ulcer (DFU). Hypoxia inducible factor 1 alpha (HIF-1α) is a significant regulator of oxygen homeostasis on hypoxic conditions. To increase our understanding of the mechanisms behind decreased wound healing in diabetes, we need to learn more about HIF-1 α regulation. Diagnosing and treating an ischemic diabetic foot is a significant issue in everyday life.

Wound healing is influenced by a variety of physiological parameters in diabetes. These include deregulation of the growth factors [3–5], macrophage functions [6], fibroblast and keratinocyte proliferation and migration, number of epidermal nerves [7], collagen deposition, quantity of granulation tissue, epidermal barrier function, [5] angiogenic response [5, 8], wound healing and control of ECM [9]. In general, clinicians caring for diabetic foot ulcer patients face the highest risks, which include failure to diagnose ischemia patients with chronic ulceration, failure to aggressively debride and treat infection, and fail to heal the wound properly. Loffler et al., [10] found that using wound fluid lactate measurement is an attractive proposition to use them as a biomarker. They discovered that high levels of wound fluid lactate should lead a physician to believe there is a risk of infection even if there are no symptoms. Subsequently, the same result revealed that there is a disadvantage, because relatively high lactate levels in non-infected diabetic wounds are most likely the result of increased tissue inflammatory activity, with leukocytes producing lactate as a by-product during their respiratory burst, rather than enhanced anaerobic energy metabolism.

Hypoxia-induced neovascularization abnormalities in the heart, skeletal muscle, nerves, and skin are all hallmarks of DM [11–13]. In diabetic individuals, insufficient collateral vessel development in response to ischemia leads to an increase in cardiovascular morbidity and death [14]. The higher prevalence of chronic wounds and lower extremity amputations in poorly treated diabetic patients is due to similar declines in compensatory neovascularization elsewhere [11, 15]. In other studies, poor production of the angiogenic cytokine vascular endothelial growth factor (VEGF) has been linked to diabetic microvascular abnormalities [16, 17]. Among the angiogenic growth factors, VEGF is unique in that it is required for both embryonic and adult vascular development. We recently discovered that defective transactivation of HIF-1 α, a master regulator that regulates the cellular response to hypoxia, results in reduced hypoxia-induced VEGF production in diabetes [18]. Under low oxygen tension, the α—subunit of this transcription factor (HIF-1) stabilises and regulates the production of numerous genes, including VEGF, that are important in the neovascular process [19]. In order to enhance disease detection and prognostic, gene profiling technology has been employed, especially in diabetes research. With the establishment of the human genome sequencing, it was discovered that the great majority of the genome is made up of non-coding specific components and non-functional segments, termed "junk DNA". The discovery of microRNAs (miRNAs), a class of tiny (22 nucleotide) single-stranded, noncoding, endogenous RNAs, has piqued researchers’ interest in recent years. Endogenously generated short non-coding RNAs (miRNAs) of about 20–22 nucleotides have been proven to have a critical role in influencing mammalian gene expression and thereby regulating various crucial physiological activities [20–24]. The rapid progress in the miRNA-based studies reflects its importance as a critical regulator of human disease and holds the promise of yielding a new class of diagnosis as well as therapeutics.

HIF-1 has previously been demonstrated to bind to a hypoxia-responsive element (HRE) on the proximal miR-210 promoter. The HRE location in the miR-210 core promoter is largely conserved across species, highlighting the role of hypoxia/HIF1-in regulating miR-210 production during evolution [25]. Thus this study focuses on HIF-1α expression and in regulating miR-210 on diabetic foot ulcers. The circulating miR-210 has been shown to be involved in epithelial cell proliferation [26], in normoxic (HIF-1α induced miR-210) differentiating myoblasts [27] as well as in Type I DM [28]. Earlier reports found that miR-210, a circulating miRNA, acts as a biomarker of breast cancer [29]. In a mouse model of ischemic wounds, hypoxia inducible miR-210 suppresses keratinocyte growth and inhibits wound healing [25]. The latest study of the miR-210 biogenesis and its physiological functions, including cell proliferation, mitochondrial suppression, arrest of DNA regeneration, vascular biometry, and angiogenesis, has also been reviewed [30–32].

Provided that miR-210 is abnormally distributed as an excellent candidate for predicting and therapeutic action, for example in several diseases such as tumor development, myocardial infarction and ischemic cutaneous (mice) skin wounds. There are hardly very few studies that looked at the role of circulating miR-210 in type 2 diabetes and foot ulcers in human subjects. Thus, this study aims in identifying its role in DFU.

## 2. Materials and methods

### 2.1. Recruitment of the study subjects

Inclusion Criteria: Healthy controls (Individuals without diabetes) and Type 2 diabetes (T2DM without any other complications) and diabetic individuals with DFU.

**2.1.1. Exclusion Criteria.** T2DM patients with other complications were excluded from the study.

All the study samples were collected as per the institutional review board- Approved protocol (Doc. Ref 005/7^th^ Feb 2013, MV Hospital for Diabetes, Chennai, SB/FT/LS-438/2012).

### 2.2. Blood samples

In this case-control study, peripheral blood samples was obtained from Control subjects (Healthy volunteers—mostly blood donors and hospital internal staffs) (Group I) (n = 50), T2DM subjects (Group II) (n = 50) and T2DM with DFU (Group III) (n = 50). All subjects were recruited from a well-established tertiary care diabetic Centre in India Chennai.

### 2.3. Diagnosis

Diagnosis of diabetes was confirmed by performing a standard 2-h Oral Glucose Tolerance Test (OGTT). Fasting plasma glucose level of ≥126 mg/dL or ≥7.0 mM after a minimum of 12 h fasting or 2-h post-glucose level [2-h oral glucose tolerance test (OGTT)] of ≥200 mg/dL or ≥11.1 mM on more than one occasion with symptoms of diabetes. Impaired glucose tolerance is defined as a fasting plasma glucose level of ≥100 mg/dL (5.6 mM) but <126 mg/dL (7.0 mM) or a 2-h OGTT of ≥140 mg/dL (7.8 mM) but <200 mg/dL (11.1 mM). Proforma on their education, occupation, smoking information, alcohol consumption, physical activity, family history was filled. All of the study participants gave their written informed consent.

The RydelSeiffer tuning fork and the 5.07 monofilament for vibration and touch were used to test peripheral neuropathy in each leg separately. In the presence of insensitivity to the 5.07 monofilament or a vibration perception of 4/8 or less, loss of protective feeling owing to diabetic neuropathy was assumed. Peripheral vascular disease is defined by an ankle-brachial pressure ratio < 0.8, the absence of ≥ 2 foot pulses or detection by means of angiography or duplex-sonography.

Purulent discharge or two or more indications of inflammation, such as warmth, soreness, swelling, and redness, are indicators of a foot infection. The location, localization, and wound condition of the ulcers were all described. The Wagner’s classification was used to assess the severity of the foot ulcers and the grade III or IV of Wagner’s was recruited for the study. The physical examinations include objective evaluation for peripheral neuropathy, the presence of other foot complications and peripheral vascular disease was performed.

### 2.4. Biochemical parameters

Biochemical parameters, as well as genomic and proteomic research, were measured using a 5-7ml venous blood sample (whole blood, plasma, and serum). The following biochemical parameters such as HbA1c, Serum lipid Profile, Plasma Glucose, total cholesterol (CHOD-PAP method), low-density lipoprotein (LDL) cholesterol, high-density lipoprotein (HDL) cholesterol, very low density lipoprotein (VLDL) cholesterol and triglycerides, total protein, albumin, bilirubin, urea, creatinine, SGOT, serum alkaline phosphatase and SGPT. All quantitative parameters are to be determined by (Dia Sys Diagnostic Systems GmbH, Germany following the Kit procedure) using a BS 400 auto-analyzer.

### 2.5. Skin specimens

Control healthy skin specimens (n = 10) was acquired as discarded tissue from control subjects who undergo surgery. During surgical debridement procedures, skin biopsies from consented foot ulcer patients (Grade IV) were taken according to an institutional review board-approved methodology. In the operating room, patients (n = 10) were debrided under supervised general anaesthesia. The non-healing edges used in this study were clinically identified by a surgeon as the most proximal skin edge to the ulcer bed. Skin biopsies were then processed as follows: (i) fixation in 4% paraformaldehyde, stored in formalin for paraffin embedding (4 μm slides) (not used in this study) (ii) stored in RNAlater (Ambion; Applied Biosystems, Carlsbad, CA) for subsequent RNA isolation and (iii) stored in laemelli buffer for proteomics studies.

### 2.6. Real time RT-PCR analysis

Initially, total RNA isolation was done by using Ambion and Invitrogen kits using their instruction manual. Quantification was done by using NanodropLite^@^NDIite, Thermo Scientific, USA. The RNA isolation was from healthy volunteers as control (Group I, n = 50), T2DM (Group II, n = 50) and T2DM with DFU (Group III, n = 50). Homogenize tissue samples (n = 10 each from control and DFU tissues) in RNAzol RT (1 mL per up to 100 mg of tissue) using an appropriate homogenizer. First strand cDNA synthesis was done by using iScript protocol (Bio Rad, USA). For Semi quantitative RT-PCR analysis, PCR primers were designed for HIF-1α gene and the expression was analyzed using semi quantitative RT-PCR using Master mix (Veriquest, USA). HIF-1 α expression was compared to beta actin expression and measured using Image Lab (Bio-Rad, USA).

Real time RT-PCR for HIF-1α mRNA was done with the Bio Rad detection system CFX96, T1000 Touch (Bio Rad, USA) using the Brilliant1 SYBR1 Green QPCR Master mix protocol (Bio Rad, USA) as well as Veriquest SYBR Green QPCR master mix (ILS, USA). Briefly, 2 μl of cDNA was amplified by PCR in 25 μl reactions containing 12.5 μl of 2 SYBR green reagents and 0.2 mM of each of the primers. The initial incubation for 10 min at 95°C was followed by 40 cycles at 95°C for 15 s and 60°C for 1 min. Triplicates of each experiment were carried out. HIF-1 expression was matched to beta actin expression.

### 2.7. miRNA isolation and expression

miRNA isolation was done by using RNAzol^@^RT (Invitrogen) from healthy volunteers as control (Group I, n = 50), T2DM (Group II, n = 50) and T2DM with DFU (Group III, n = 50) serum as well as tissues from control (n = 10) and DFU (n = 10).The first strand cDNA was synthesized from 10 ng of the miRNA using First-strand cDNA Synthesis Kit and microRNA- specific RT primer sets for target miRNA (hsa-miR- 210) and endogenous control miR16 (Exiqon).miRNA expression was analyzed by Quantitative Real Time PCR analysis. The analysis was performed using mercury LNATM microRNA PCR system, SYBR green master mix and with LNA-based primer sets for target miRNAs (hsa-miR-210) and miR16 as an endogenous control (has-miR-16) (Exiqon). The expression level of miRNA was determined using 2-ΔΔ Ct and normalized to miR16 and represented as fold change.

### 2.8. ELISA and Western blot

The levels of HIF-1α, VEGF, TNF-α and IL-6 were screened in the serum of the study groups by using commercially available ELISA kits (Peprotech, USA). Tissues collected were homogenized with ice-cold homogenizing buffer (50mM Tris-HCl, 150mM NaCl, 1mM EDTA, and 0.5mM Triton X-100, PH 7.4) and protease inhibitor cocktail tablets (Roche, Germany). Proteins were measured with Bio-Rad protein assay method. DFU and control tissues protein lysates (50μg/well) were separated by SDS-PADE (12.5%) under reducing conditions and transferred to a polyvinylidenedifluoride (PVDF) membrane (Millipore, USA). Membranes were treated with Bio Rad western blot system kit, according to the manufacturer protocol (Biorad, USA). Briefly, blots were blocked with blocking buffer (5% not-fat dried milk in PBS). After blocking, blots were incubated with anti-Bcl-2 polyclonal antibody (1/500, v/v), anti-HIF -1 alpha monoclonal antibody (1/500, v/v), anti- caspase 3 antibody (1/500, v/v) and anti-Bax polyclonal antibody (1/250, v/v) (Santa Cruz, Biovision, USA) for overnight at 4°C. Blots were washed for 4 times with 0.1% tween 20 in PBS and incubated with HRP conjugated secondary antibody (1/5000, v/v, Biochain, USA) for 1 hour at room temperature. The HIF- 1α, Bcl-2, Bax and caspase 3 protein bands were visualized using enhanced chemiluminescence (ECL) method (Bioimaging, system, UK).

### 2.9. Statistical analysis

Statistical analysis was done using SPSS 7.5 version, USA. The data were analyzed by one-way ANOVA followed by Tuckey’s multiple range test to assess the significance (P<0.05 considered significant).

## 3. Results

### 3.1. Clinical characteristics of the study subjects

The clinical parameters such as BMI, fasting glucose, post prandial glucose and HbA1c were significantly high in T2DM and DFU when compared to that of control subjects. HDL cholesterol was significantly low on T2DM and DFU when compared to that of control subjects. However, other parameters such as age, total serum cholesterol, urea and creatinine didn’t show any significant differences between groups (Table 1).

**Table 1:** Biochemical parameters of the study subjects.

N	Variables	Healthy controls (N = 50)	T2DM (N = 50)	T2DM with DFU (N = 50)
1	Age (Years)	48± 10.46	52 ± 11.36	53 ± 11.24
2	Fasting blood glucose (mmol/l)	5.7±0.6	10±4.7**	11±4.6**
3	Post prandial blood glucose (mmol/l)	6.6 ±0.8	11.6±4.5**	12± 4.5**
4	Glycosylated Haemoglobin (mmol/mol)	37.7±4.5	69.4 ±7.1***	83.6±8.0***
5	Total cholesterol (mmol/l)	9.6 ±1.8	10.4± 3.2	9.9±4.3
6	HDL cholesterol (mmol/l)	1.21±0.2	0.7±0.05*	0.75±0.1*
7	Triglycerides (mmol/l)	6.7± 2.5	8.5±3.2	8.4± 4.2
8	Plasma Urea (mmol/l)	1.3±0.4	1.4±0.9	1.9± 1.1
9	Plasma Creatinine (μmol/l)	49± 4.5	53± 4.8	54±5.9

This Table represents clinical and biochemical characteristics of different study subjects.

*Represents the significance at the level of P < 0.05 when compare to that of healthy controls.

**Represents the significant at the level of P < 0.01 when compare to that of healthy controls.

***Represents the significant at the level of P < 0.001 when compare to that of healthy controls.

### 3.2. Serum expression of HIF-1α and miR-210 on study subjects

**Fig 1:**
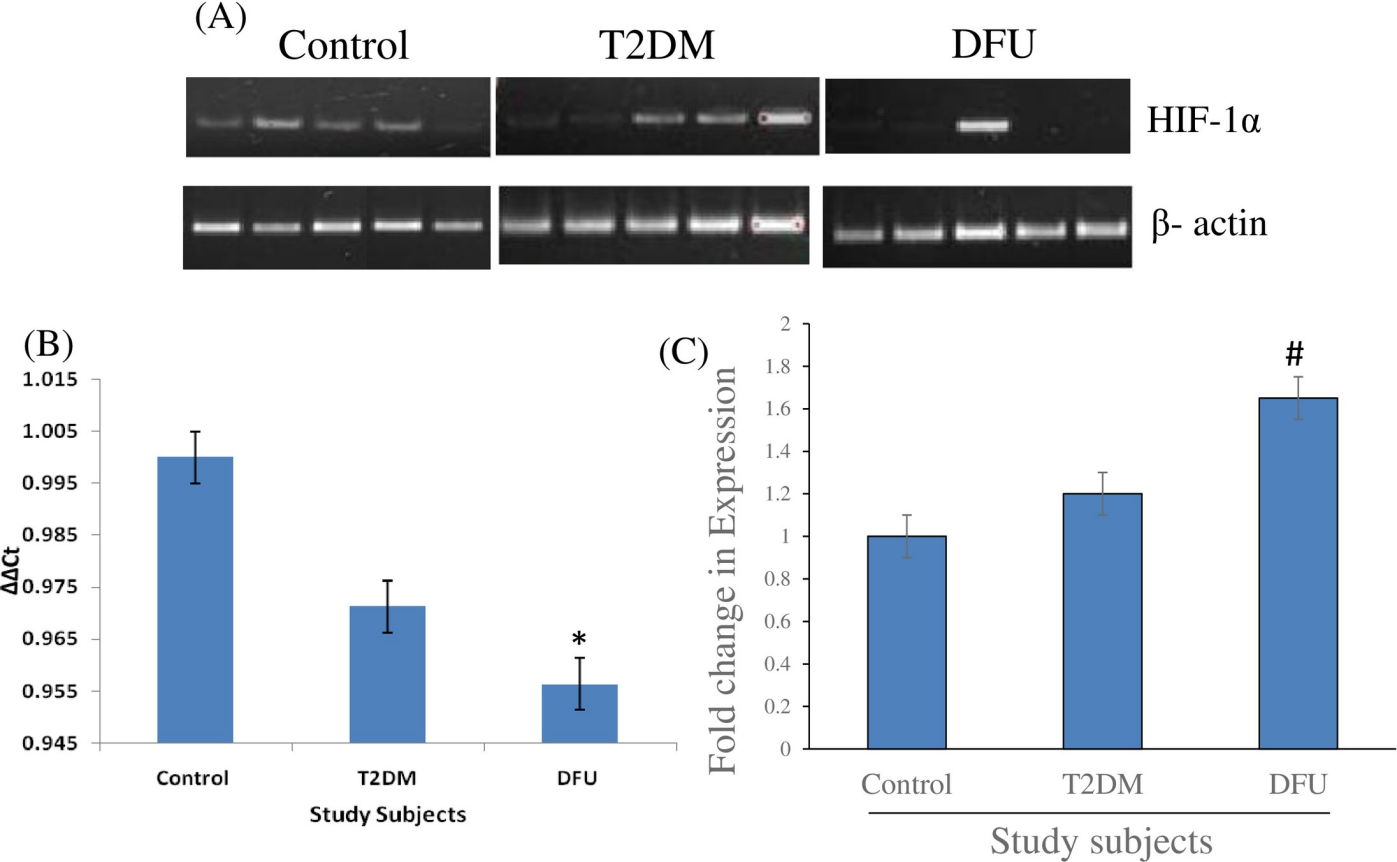
miR-210 and HIF-1α mRNA expressionin serum of study subjects. (A) Semi-quantitative RT-PCR was performed as described in materials and methods. The gel image is showing the expression pattern of HIF-1α and β-actin on different study subjects. (B) HIF-1α mRNA expression from serum of study subjects, analyzed by quantitative RT-PCR. In the above experiments, the relative expression of HIF-1α mRNA was calculated after normalization with β-actin mRNA. (*) indicates significant decrease compared to control/healthy or T2DM sample. (C) miR-210 expression in serum analyzed by quantitative RT-PCR. miRNA isolation and quantitative RT-PCR analysis was carried out as described in materials and methods. The relative expression of mature miR-210 was calculated after normalization with miR-16. (*) indicates significant increase compared to control/healthy or T2DM sample.

To investigate the expression of HIF-1α in serum of study subjects the semi quantitative RT-PCR analysis was performed initially. The result showed that the HIF-1α expression was drastically decreased in DFU subjects when compare to that of diabetes subjects. The expression pattern is normalized with the house keeping gene β-actin (Fig 1A) [33]. Further, it was analyzed by real time RT-PCR analysis.

The results confirmed that the decreased expression of HIF-1α was noted on foot ulcer patients when compare to that of diabetic subjects and the expression levels were normalized with that of housekeeping gene β-actin. The results were much correlated with that of semi-quantitative RT-PCR results (Fig 1B). Similarly, to understand miR-210 expression pattern in the serum of study subject, miRNA was isolated and real time rime RT-PCR analysis was performed as described in materials and method section. The results showed that increased expression of miR-210 was noted on foot ulcer patients when compare to that of diabetic subjects and the expression levels were normalized with that of miR-16 which constantly expressed in all tissues (Fig 1C). Altogether, the results showed an inverse correlation between miRNA-210 and HIF-1α expression in the study subjects.

### 3.3. Tissue expression of HIF-1 α and miR-210 on study subjects

**Fig 2:**
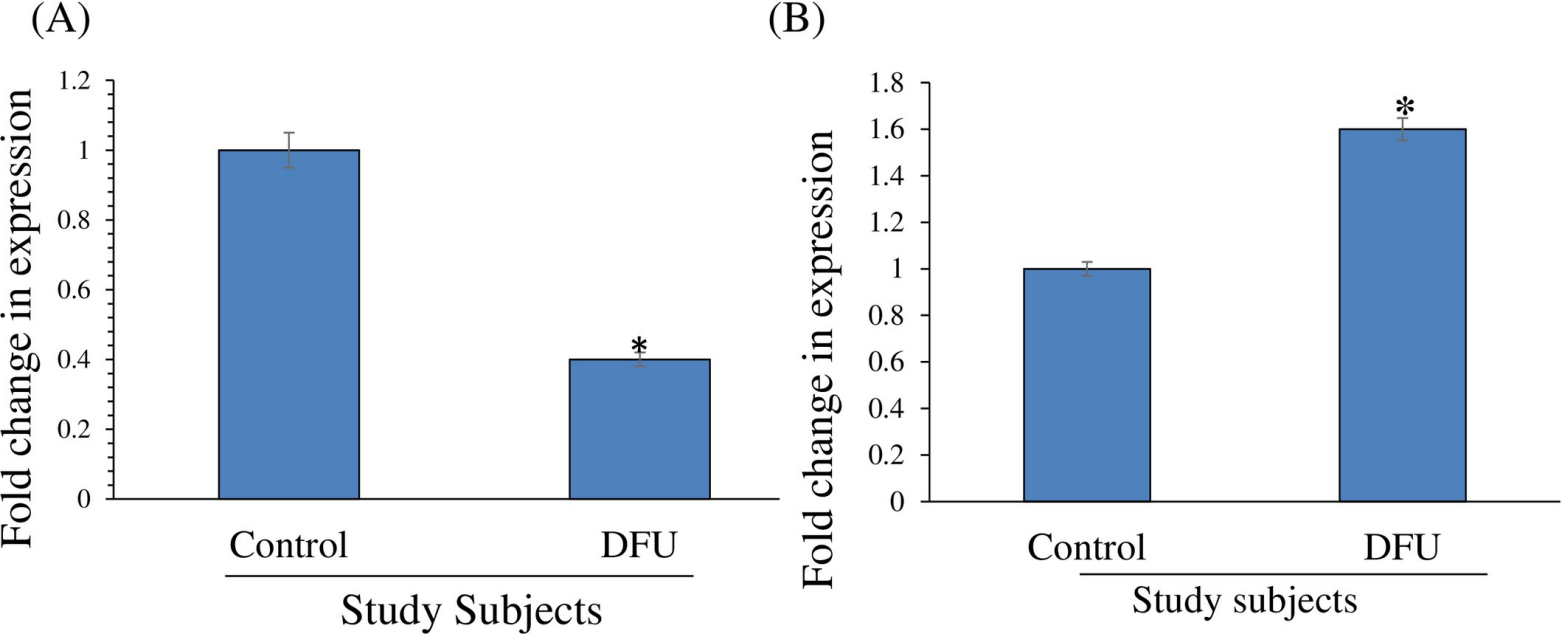
miR-210 and HIF-1α mRNA expressionin tissue of study subjects. (A) Total RNA was isolated from control and DFU tissue. Quantitative RT-PCR analysis was carried out for HIF-1α mRNA expression. Fold change of HIF-1α mRNA expression was calculated after normalization with β-actin mRNA. (*) indicates significant decrease compared to control tissue. (B) Quantitative RT-PCR analysis was carried out for miR-210 expression. Fold change of miR-210 expression was calculated after normalization with miR-16. (*) indicates significant decrease compared to control tissue.

The expression pattern of miR-210 and HIF-1α was analyzed in serum of study subjects (Fig 1). However, to understand their expression pattern in tissue samples total RNA was isolated and real time RT-PCR analysis was carried out. The results documents that there was decreased expression of HIF-1α on the tissues of foot ulcer patients when compare to that of control tissues and the expression levels were normalized with that of housekeeping gene β-actin (Fig 2A). In a similar manner, miR-210 was extracted and real-time RT-PCR analysis was carried out, in conjunction with the materials and methods section. The results confirmed that there was increased expression of miR-210 on the tissues of foot ulcer patients when compare to that of control tissues and the expression levels were normalized with that of miR-16, a constituently expressing gene (Fig 2B).

### 3.4. Protein expression levels of HIF-1α on different study subjects

**Fig 3:**
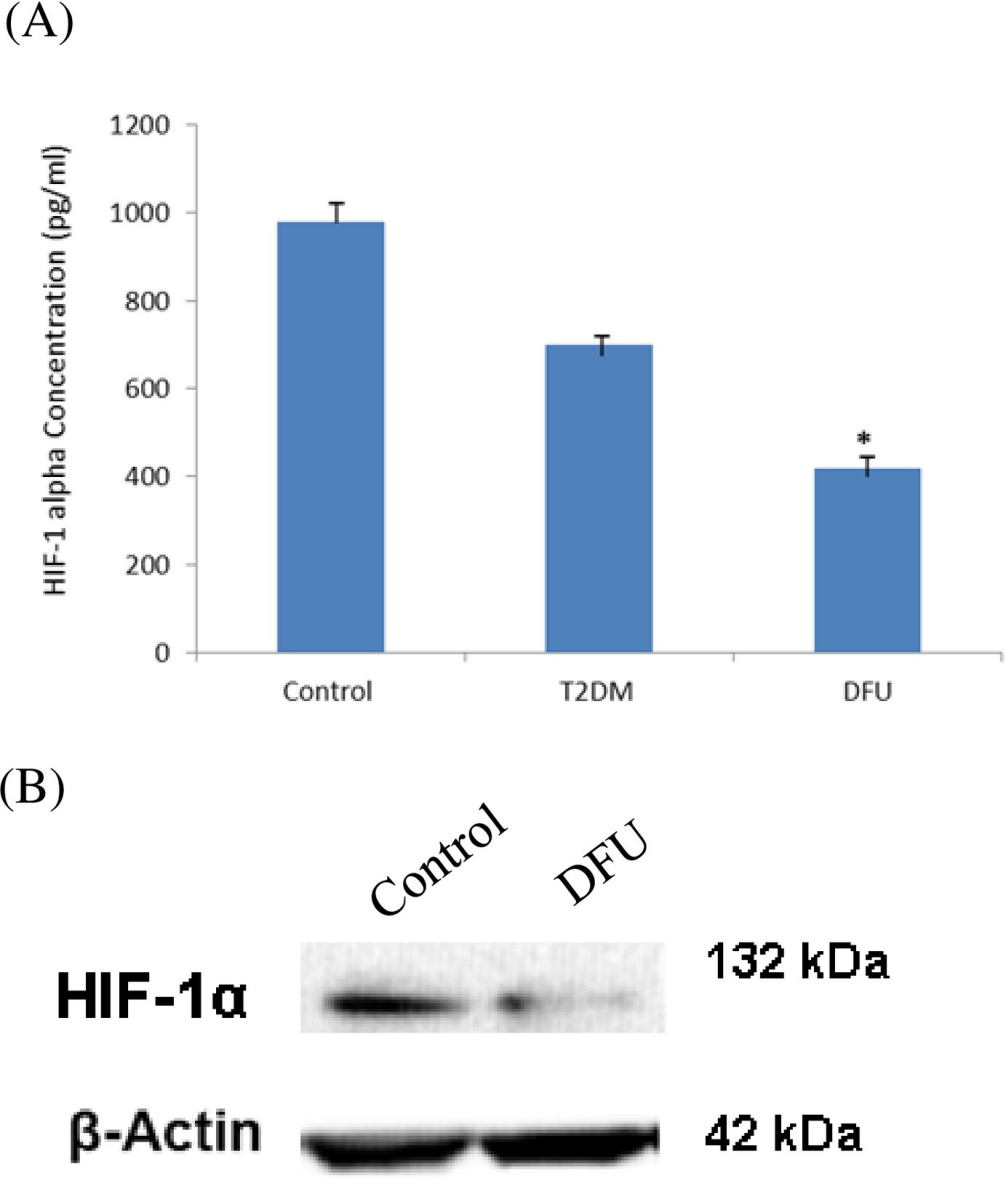
HIF-1α protein expression from serum and tissue of study subjects. (A) Serum was obtained from T2DM, DFU and healthy patients and total serum protein was extracted, and HIF-1α expression was analyzed by ELISA. (*) indicates significant decrease compared to control or T2DM. Similarly, (B) Total protein was extracted from tissue of control and DFU samples and Western blot analysis of HIF-1α expression was performed.

The transcript level of HIF-1α expression from different study subject was analyzed (Figs 1 and 2). Additionally, transcriptome levels of HIF-1α expression were analyzed. The serum protein expression levels of HIF-1α is done by ELISA (Fig 3A) and the results showed significantly decreased expression was seen in DFU patients (P<0.005) when compared to that of T2DM and Control subjects (n = 50 each) (Fig 3). In addition, Western blot study was carried out on the tissue of study subjects and the results showed reduced production of HIF-1α on DFU when compare to that of control tissues. (Fig 3B). In both serum and tissue of the study subjects, the same common pattern of HIF-1α protein expression was observed (Fig 3A and 3B).

### 3.5. Circulatory protein expression of hypoxic downstream signaling targets

**Fig 4:**
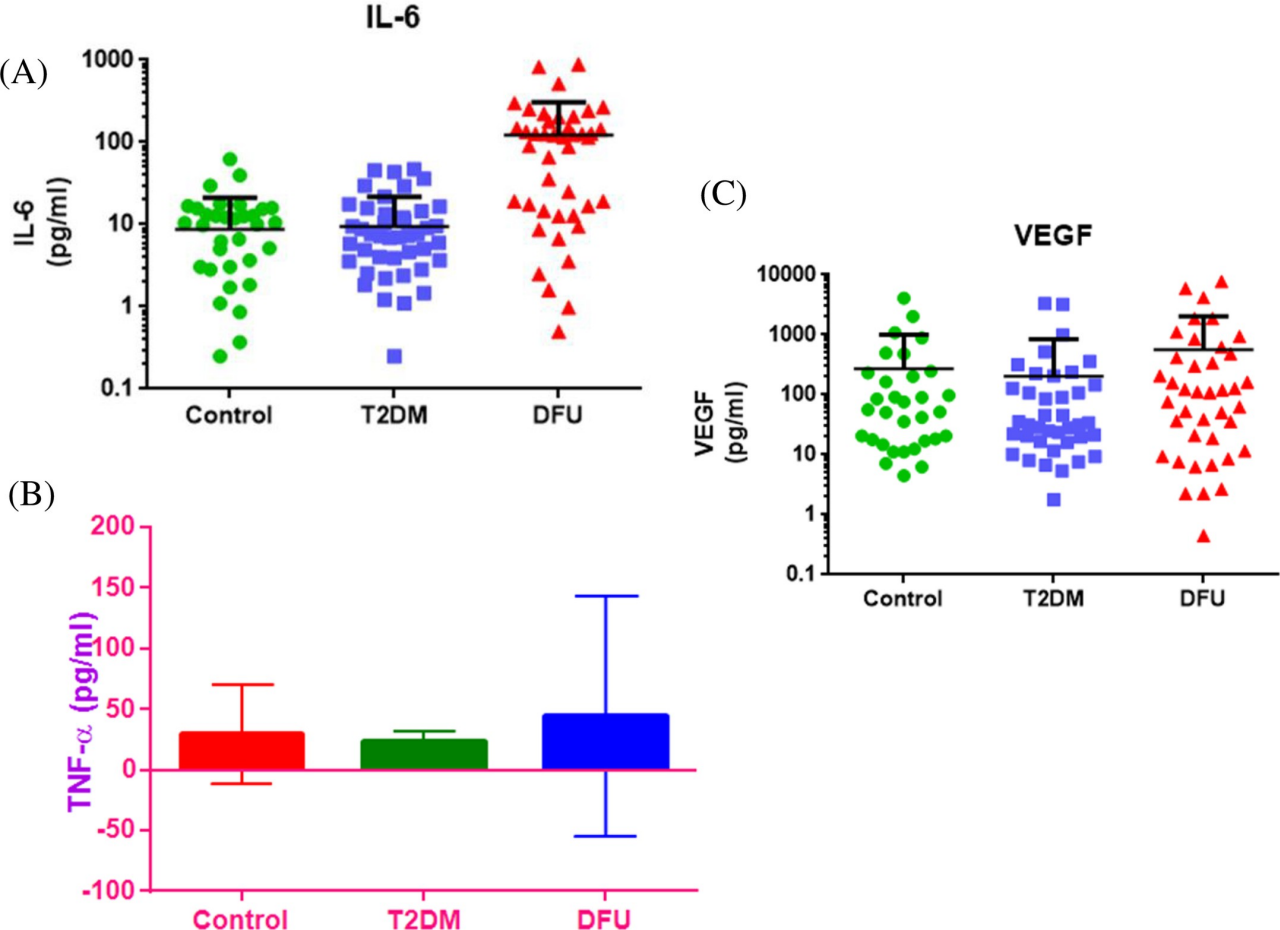
Hypoxic downstream signaling molecules, IL6, TNF-α& VEGF analysis from study subjects. Serum protein was isolated from control, T2DM and DFU patients and IL6 (A) and TNF-α(B)and VEGF (C) were quantified by ELISA.

The findings (Figs 1 and 3) revealed that the HIF-1α was distributed significantly in patients with DFU and, in particular, that miR-210 was reversed in HIF-1α expression. Hence, it is required to understand the hypoxic downstream signaling targets from the same study subjects. In this case, differences in translational expression of hypoxic downstream signaling targets such as IL6, VEGF and TNF-α were analyzed on different study subjects using ELISA. Reports suggested that there was increased expression of IL6 (Fig 4A) and TNF-α (Fig 4B) on DFU subjects when compared to that of T2DM and control subjects but not statistically significant. Additionally, VEGF, an angiogenic molecule was also analyzed but there was no significant differences observed between the study subjects (Fig 4C).

### 3.6. Tissue expression of apoptotic proteins

**Fig 5:**
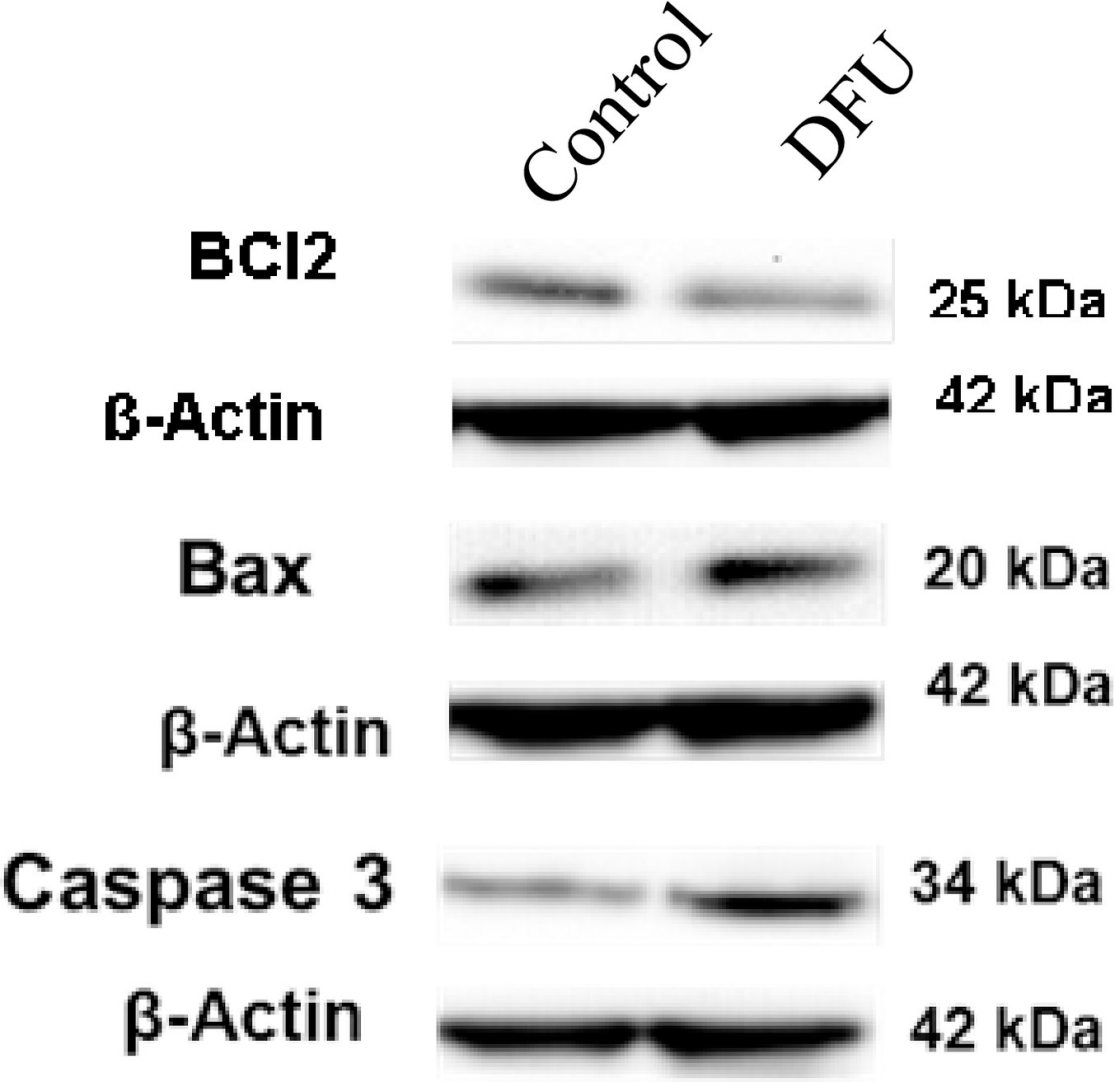
Apoptotic proteins, BCl2, Bax and Caspase 3 expression in tissue of study subjects. Whole cell lysates were prepared from control and DFU tissues and Western blot analysis was performed using the antibodies as indicated in materials and methods; β-actin was used as internal control.

The role of oxidative stress, which leads to increased lymphocyte death, may be important in the delay of wound healing. In this study, Caspase 3, which is the most important of the executioner Caspases was studied and it was found to be higher in DFU patients when compare to that of control subjects (on Western blot analysis). BCl2 proteins can promote or inhibit apoptosis by direct action of Bax. We found that decreased BCl2 with increased BaX on DFU patients when compare to that of control subjects (Fig 5).

## 4. Discussion

DM is a serious systemic disease and is increasing worldwide vastly. Lower extremity manifestations are mostly associated with morbidity and mortality. Diabetic foot disease arises from chronic pathologic process such as neuropathy, peripheral artery disease and impaired wound healing. Diabetic foot disease is a major health problem, which affects 15% patients with diabetes worldwide [31]. Slow or non-healing diabetic wounds are the major cause of non-traumatic lower limb amputation in underdeveloped nations. As a result of the impaired blood vessel creation in reaction to ischemia, these wounds heal slowly. As a result, a deeper knowledge of the molecular mechanisms underlying the increased rates of problems in diabetic patients is critical in order to create more effective medicines and early detection.

The oxygen content at the wound site is the most important factor in wound healing. The transcriptional stimulation of a variety of genes involved in angiogenesis, iron metabolism, glucose metabolism, and cell proliferation/survival occurs when cells and tissues adapt to low oxygen tension (hypoxia). The primary factor mediating this response is the HIF-1α, an oxygen-sensitive transcriptional activator [18]. Chemokines are important for blood cell recruitment to the location of wounded tissues, as well as during wound healing [32]. Earlier studies in our lab showed that decreased genetic expression of HIF-1α was observed in DFU patients when compare to that T2DM and control subjects on pro582 ser polymorphic studies [33]. The same trend was observed in circulatory protein and the expression of HIF -1α protein expression was decreased in DFU when compare to that of T2DM and control subjects on exon 12 of HIF-1α mutation subjects [34].

MiRNAs are involved in numerous biological processes, including proliferation, differentiation, apoptosis, and development, and there is a plethora of evidence demonstrating their importance in a variety of diseases and disorders. The list of disorders with which miRNA dysregulation has been linked is constantly rising [35, 36]. The significance of miRNAs in diabetes and associated consequences is significantly less understood than it is in cancer. Several miRNAs have been discovered to play a physiological role in tissues that are affected by diabetic problems. It’s yet unclear if these miRNAs play a role in the damage caused by diabetes. Because miRNAs have the ability to modulate key physiological processes and pathophysiological disease states, identifying the miRNAs and their targets associated with diabetic complications (such as foot ulcers) is critical, as it will open up a new window of opportunity for discovering new biomarkers and therapeutic targets.

MiRNAs may meet a major demand for biomarkers for the early detection of diabetic problems, as they are generally stable and easily quantified in a non-invasive manner in plasma and urine. This would considerably improve clinical management of long-term outcomes. Recent research has shown that miRNAs have a role in the control of gene expression in a variety of skin cells, including stem cells, immune cells, and keratinocytes [37]. Keratinocyte migration, proliferation, and differentiation are all important steps in wound healing because hair follicle keratinocytes (initially) and epidermal keratinocytes (later) finally fill up the wound gap and restore skin integrity. Earlier studies have showed that miR-210 promote keratinocyte differentiation [38] and are upregulated in diabetic mice and is upregulated in both T1DM and T2DM patients [39]. Earlier studies showed that there is a inter relationship between miR-210 and HIF 1 alpha and the studies showed that miR210, the fine tuner on the hypoxic response [40]. The HIF-1α and its miRNA target, miR-210, are candidate tumor-drivers of metabolic reprogramming in cancer [41]. There are no studies till date for the association of HIF-1α dependent miR-210 regulation on DFU. In this study we observed that miR210 was over expressed with the decreased expression of HIF -1α on circulation and in foot ulcer tissues when compare to that of control subjects. We identified the gene expression pattern of hypoxic gene (HIF-1α) and its regulatory micro RNA (miR-210) and its effect on downstream proteins. HIF-1α gene expression is significantly decreased in foot ulcer patients when compared to that of diabetic subjects and healthy controls, whereas miR-210 expression is increased in foot ulcer subjects when compare to that of healthy controls and T2DM. This study showed that miRNA expression (miR-210) is reversibly proportional to that of hypoxic gene HIF-1α. We observed that the downstream proteins (such as IL-6) mediated by HIF -1α were found to be significantly increased in foot ulcer patients when compare to that of healthy controls and T2DM subjects, whereas other inflammatory and angiogenic markers such as TNF-α and VEGF expression showed no significant difference between the groups. Earlier reports showed the effect of different angiogenic factors and its expression on diabetic wound healing [42–45]. The tissue as well as circulatory expression of HIF-1α and miR-210 was observed between different study subjects. The results showed same correlation between tissue and the circulatory levels of miR-210 and HIF-1α, which indicates that this may use as a diagnostic biomarker in future for preventing the amputation on diabetic patients.

In this study we found that HIF -1α is directly involved on apoptosis pathway. The differential expression of apoptotic markers such as Caspases, Bax, BCl2 were observed on DFU tissues when compare to that of control subjects. These observed findings may be due to the miR-210 over expression caused significant inhibition of the HIF-1α pathway and attenuated apoptosis caused by hypoxia. Together our findings showed that miR-210 is involved in the molecular response on diabetic foot tissues and attenuated hypoxia induced cell apoptosis by targeting HIF -1α directly and suppressing HIF-1α downstream pathway activation. Thus, our findings together suggest that association of HIF-1α dependent miR-210 regulation on DFU, and it may use as a potential circulatory diagnostic biomarker in future.

## Supporting information

S1 Data (PPTX)
