# Supplementary material for: Impact of microRNA-210 on wound healing among the patients with diabetic foot ulcer
Source: PLoS One. 2021 Jul 22;16(7):e0254921. doi: 10.1371/journal.pone.0254921 (PMC8297780; doi:10.1371/journal.pone.0254921)
Supplement: S1 Data — (PPTX) [file pone.0254921.s001.pptx]

## Slide 1
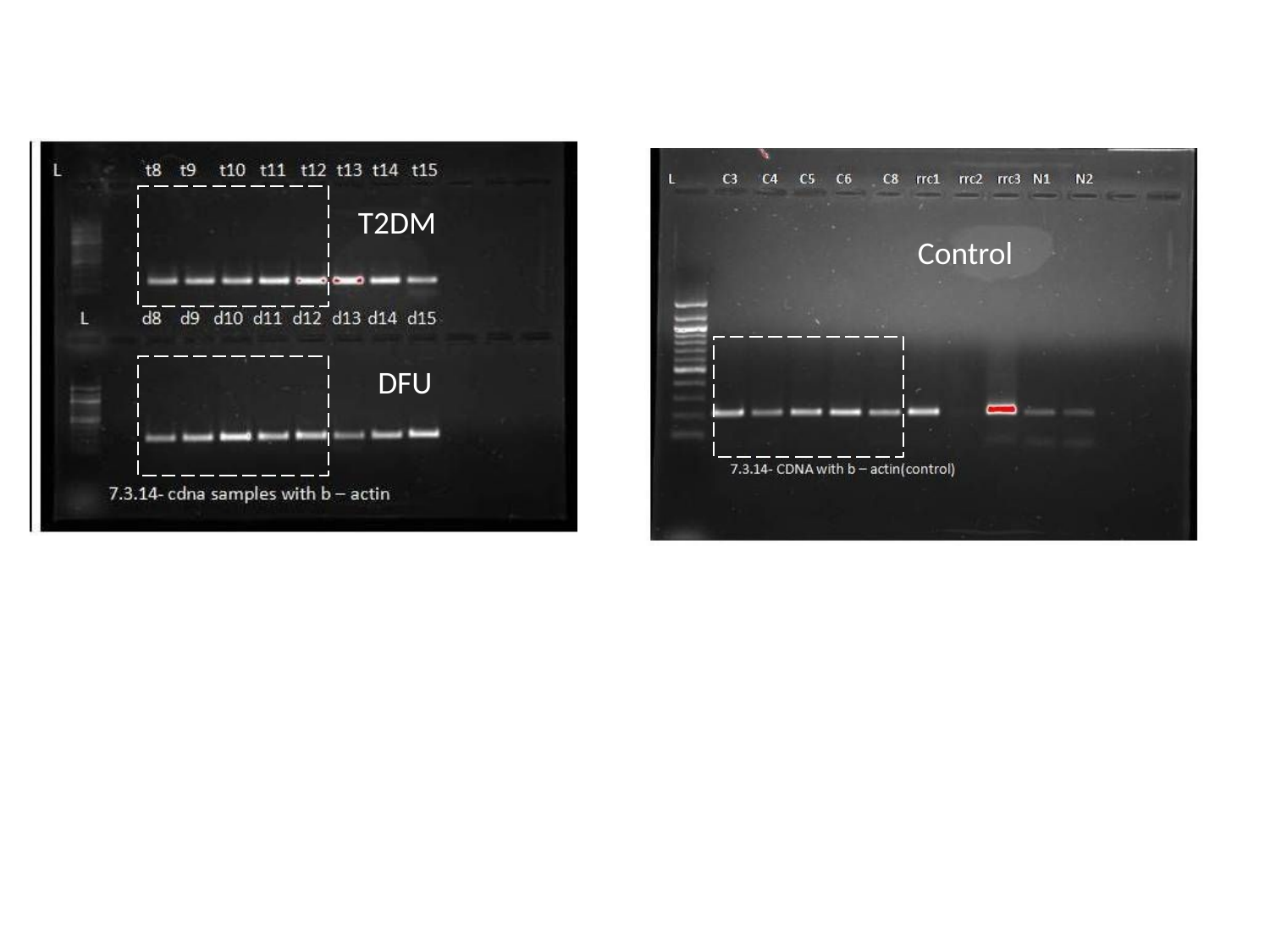

T2DM
Control
DFU

## Slide 2
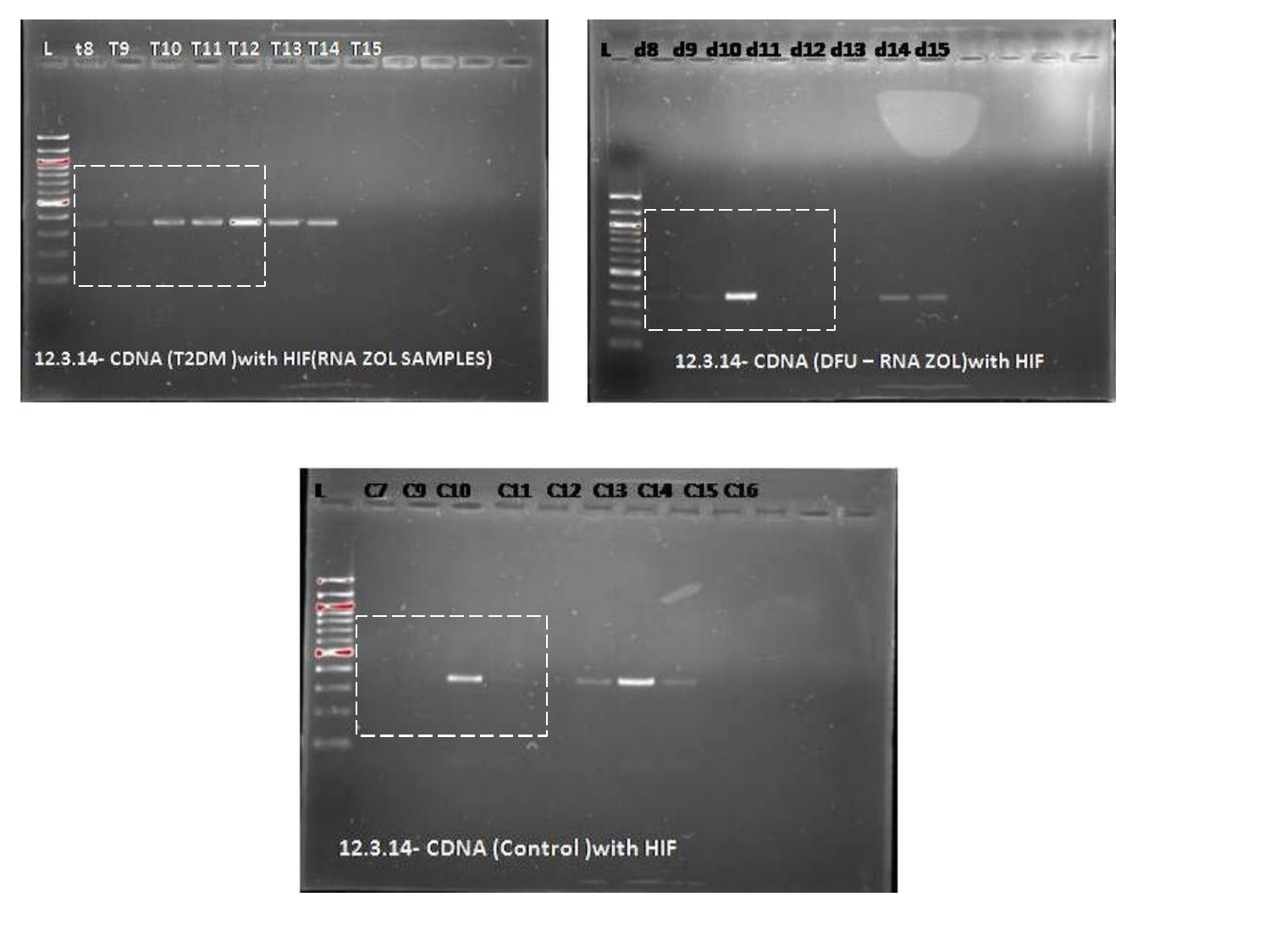

## Slide 3
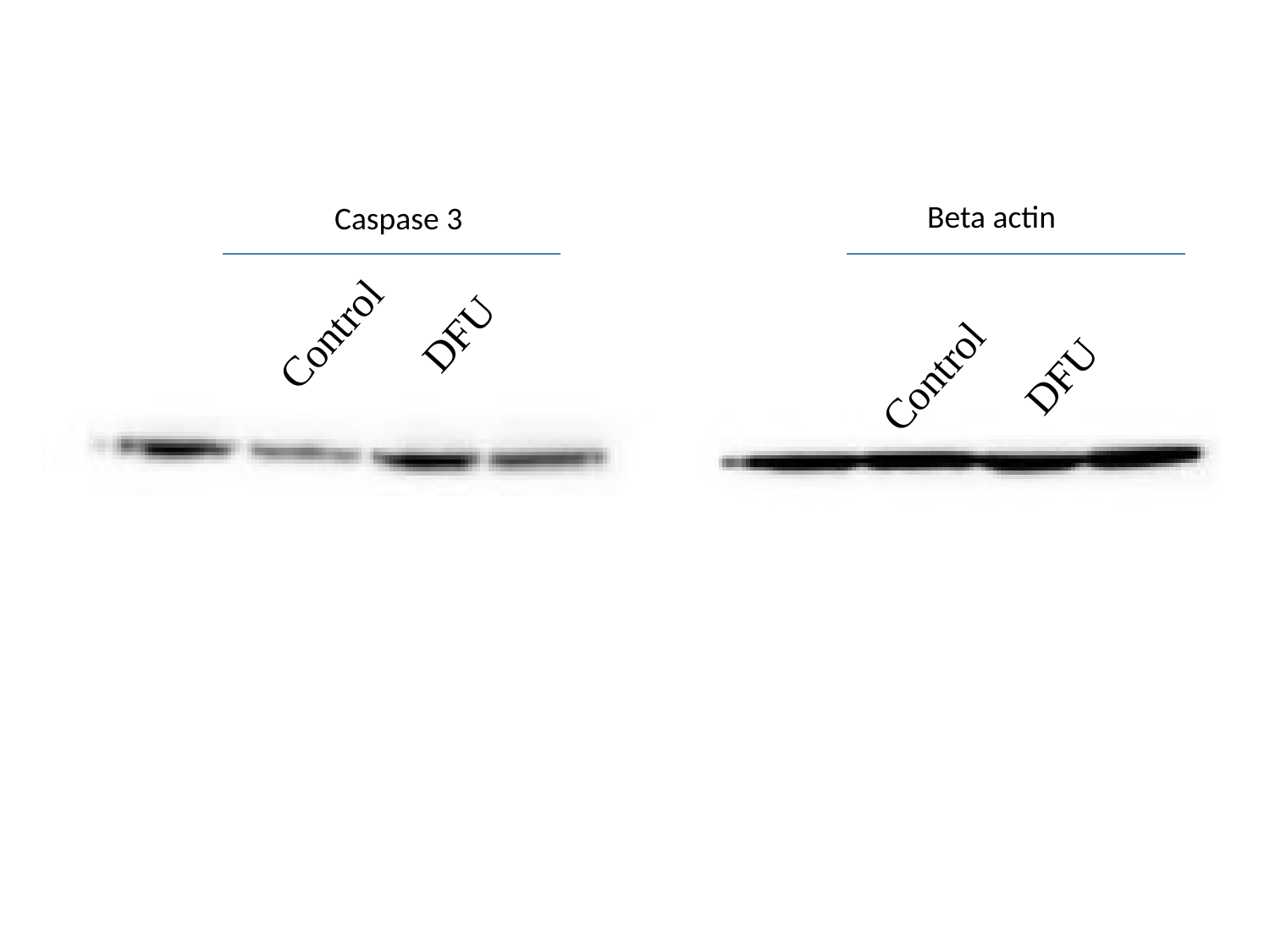

Beta actin
Caspase 3
Control
DFU
Control
DFU

## Slide 4
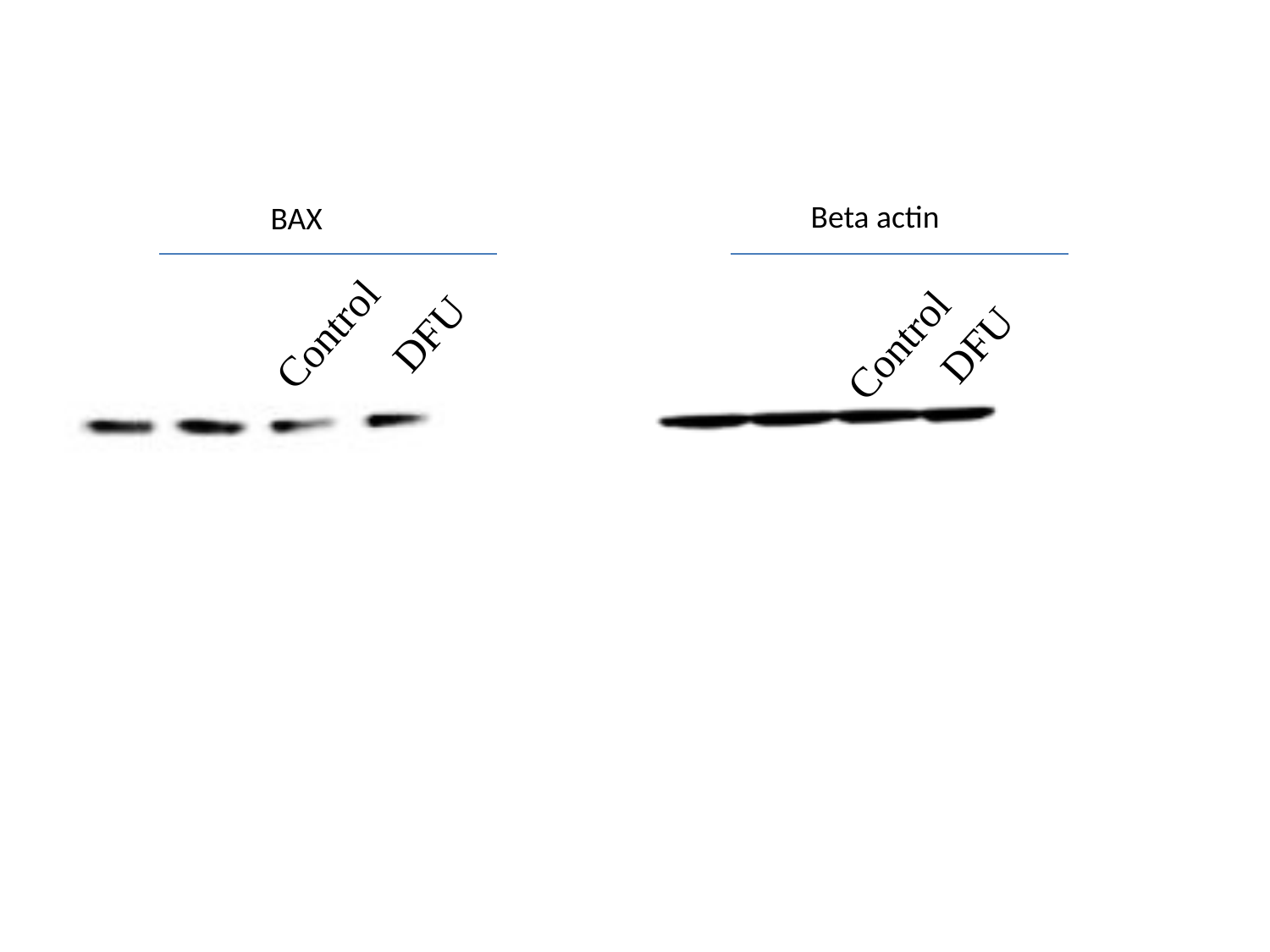

Beta actin
BAX
Control
DFU
Control
DFU

## Slide 5
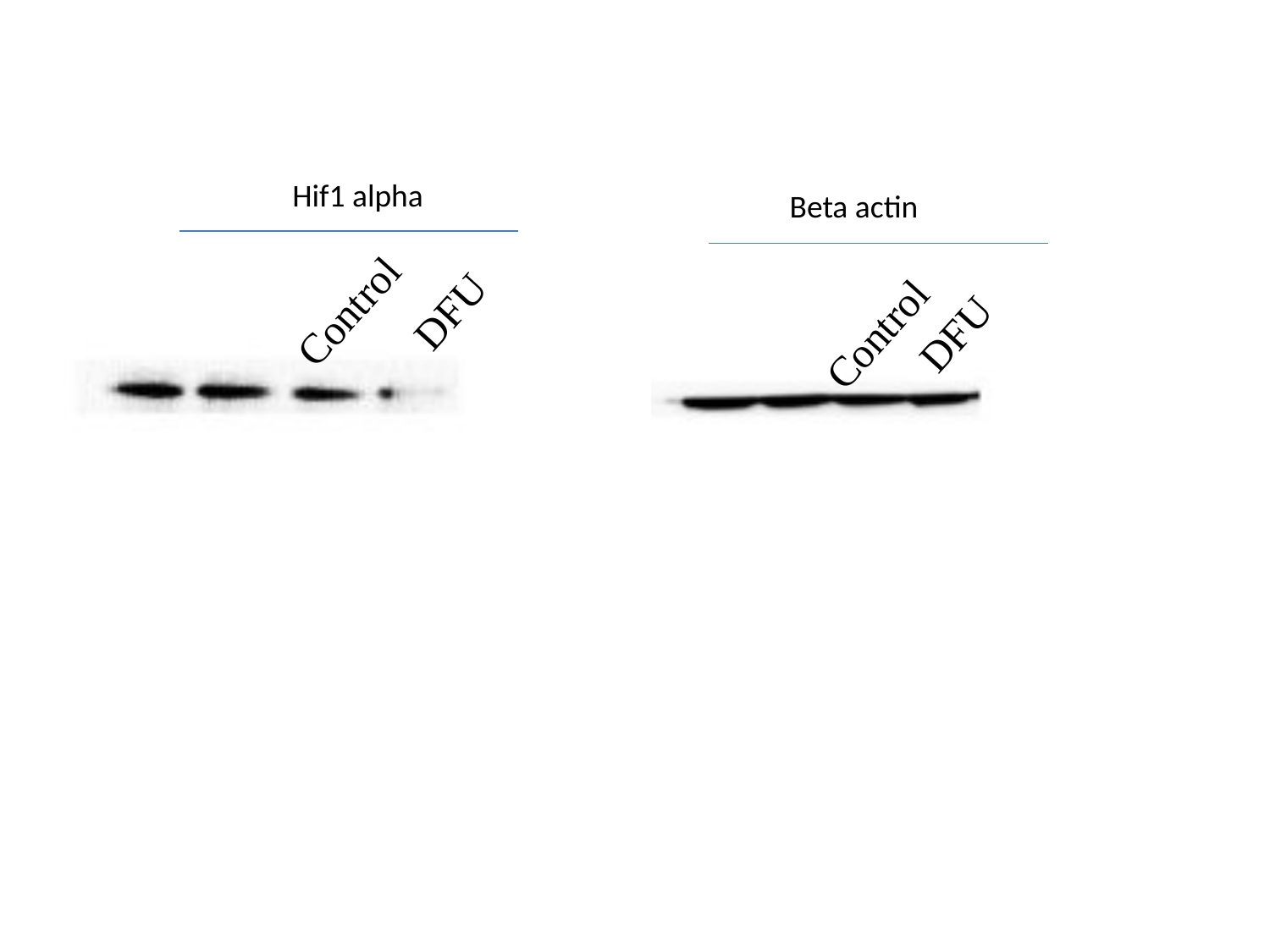

Hif1 alpha
Beta actin
Control
DFU
Control
DFU

## Slide 6
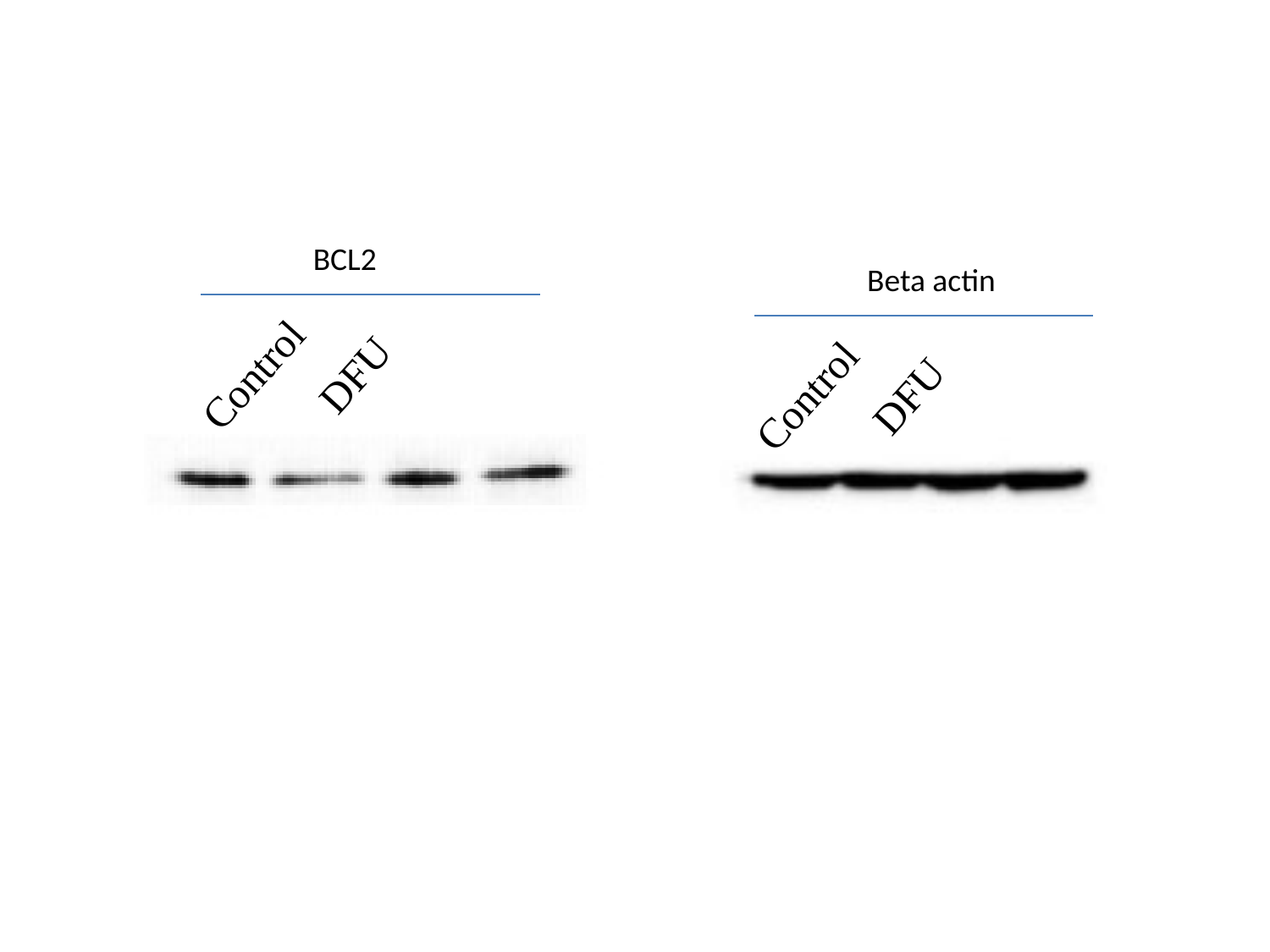

BCL2
Beta actin
Control
DFU
Control
DFU
